# Medical Opinion and Movement

**Published:** 1908-02-08

**Authors:** 


### Medical Opinion and Movement.

Some years ago Freund expressed the opinion that a certain number of cases of emphysema originate in alterations in the cartilages of the ribs causing a fixation of the chest in a condition of inspiration, and he suggested that the condition might be relieved by operation. The costal cartilages become thickened and deformed, and on section are of a yellow colour and are riddled with cavities, their elasticity being completely lost. By division of the affected cartilages, it was argued, the mobility of the chest would be restored, and the ribs would subside during expiration. These suggestions were not put into practice till 1906, when Freund published his first case. Since then several further cases have been published, and in each considerable relief has been afforded by the operation. Professor Lejars, in drawing attention to the whole subject, points out that it is too early to form any conclusion as to the permanent results of the operation, but it offers a prospect of relieving cases which are otherwise hopeless. It is, of course, only applicable to cases of emphysema which present a fixed dilated barrel-shaped thorax, with the peculiar changes in the cartilages. The operation should be performed before grave organic changes have taken place in the lungs and heart.

So long as the etiology of a disease has not been established on a firm basis by proved facts, any theory which has a semblance of probability deserves consideration, the more so when it offers a working hypothesis for a definite line of treatment, which can be shown to be at least efficacious in certain cases of the disease. Quite recently we reviewed the theory put forward by Professor Ehrlich that the parasyphilitic diseases, locomotor ataxia and general paralysis are the result of an excessive immunisation of the central nervous system against the syphilitic virus. Dr. Le Grand Winslow, of New York, suggests that locomotor ataxia is due to an irritation of the peripheral nerves, and that this irritation, causing reflex troubles in the spinal cord and brain, by persisting sufficiently long, determines the pathological changes in the spinal cord characteristic of the disease. He explains the supposed connection between the disease and syphilis by supposing that the syphilitic virus renders the nervous system susceptible of such degenerative changes by irritation. Such a theory is not therefore at all incompatible with the hypothesis set forth by Professor Ehrlich. Dr. Le Grand Winslow gives details of twelve cases in which he has located the supposed peripheral irritation as a urethral stricture, and in which treatment of the stricture by dilatation has been followed by total arrest of the disease, and in some cases by marked improvement, with total disappearance of some of the symptoms. Power of co-ordination has returned, lightning pains have disappeared, and even the pupils have regained the power to react to light. Such results are certainly remarkable, and give encouragement for further work on the same lines.

In view of the conflicting opinion of the medical profession not only in regard to the consumption of alcohol in health, but also in respect of its therapeutic value in disease, the inquiry which has been started by the International Union of Medical Abstainers should prove to be of considerable utility. This International Union came into existence last year at Stockholm, and as part of its programme of work it has undertaken an inquiry into the value of alcohol in lobar pneumonia and typhoid fever, with the hope of obtaining evidence by which a definite and authoritative opinion can be formed on the subject. The staffs of the hospitals and infirmaries of the countries in which the Union is represented have been invited to co-operate on one of two plans. One of them consists in furnishing a return of the results of treatment of cases of these two diseases whether by alcohol or otherwise. The second plan is definitely to treat all cases either of the one disease or the other with or without alcohol alternately. Dr. J. J. Ridge, of Carlton House, Enfield, is the representative of the Union in this country. Such a plan should eliminate personal prejudice and opinion, and afford a broad basis for accumulating facts and statistics on the question. There is perhaps no point in regard to the utility of alcohol which has been more hotly contested than its value in the treatment of these two diseases. A clear verdict based on incontrovertible evidence is therefore most desirable.

Professor Francis Valk, of New York, has recently reported on the extremely interesting operation of grafting a rabbit's cornea on the human eye. The author believes the operation was originally devised by Reisinger, but it was first successfully performed by Von Hippel in 1887. Since then he finds no record of any cases apart from his own two cases which were reported in 1889. The third operation was carried out last year. Suitable cases for the operation are extremely rare. He sums up the condition thus: a total leucoma without complications in the posterior surfaces of the cornea or in the iris. Unfortunately cases of leucoma resulting from ophthalmia neonatorum or a gonorrheal infection later in life almost invariably present an infiltration of the deeper tissues, involving Descemet's membrane, and generally the iris also, and in such cases the operation would, of course, be useless. In regard to the operation itself, the questions which first present themselves are, Will the graft live? and Will it remain transparent? In each of the three cases the graft has lived and has seemed to become a permanent part of the cornea. In the first case the graft became so opaque that no improvement was obtained in visual power. In the second case the graft became only partially opaque, and the visual power was decidedly improved, so that the patient was able to move about and do simple housework. In the third case also, up to the time of reporting, the graft was in good condition and fairly transparent. In suitable cases and in proper hands the operation offers some measure of success, and therefore seems entirely justifiable for those who have lost all useful vision.

